# Effects of Gap 26, a Connexin 43 Inhibitor, on Cirrhotic Cardiomyopathy in Rats

**DOI:** 10.7759/cureus.59053

**Published:** 2024-04-26

**Authors:** Dlshad Mohammed, Seyed Mohammad Tavangar, Arash Khodadoostan, Seyyedeh Elaheh Mousavi, Ahmad-Reza Dehpour, Farahnaz Jazaeri

**Affiliations:** 1 Pharmacology, Tehran University of Medical Sciences, Tehran, IRN; 2 Pathology, Tehran University of Medical Sciences, Tehran, IRN; 3 Pharmacology, Shahid Beheshti University of Medical Sciences, Tehran, IRN

**Keywords:** cirrhotic cardiomyopathy, connexin 43, gap 26, inflammation, oxidative stress

## Abstract

Introduction

Cirrhotic cardiomyopathy (CCM) is recognized by impaired cardiac responsiveness to stress, prolonged QT interval, and systolic and diastolic dysfunctions. Connexins are a family of transmembrane proteins that play a key role in cardiac physiology. Connexin 43 (Cx43) inhibition showed cardio-protective effects. Peptide drug Cx43 inhibitor, Gap 26, could inhibit gap junction 43. This study was designed to evaluate the effects of a connexin mimetic peptide, Gap 26, in the CCM model in rats.

Methods

The cirrhosis was induced through carbon tetrachloride (CCl4). On day 56, electrocardiography (ECG) was recorded, spleen weight was measured, and tissue and serum samples were collected. Further, Cx43 mRNA expression in heart tissue was checked.

Results

The chronotropic responses decreased in the CCl4/saline and increased in the CCl4/Gap. The spleen weight, QTc interval, and brain natriuretic peptide (BNP), tumor necrosis factor-alpha (TNF-α), aspartate aminotransferase (AST), alanine transaminase (ALT), and malondialdehyde (MDA) levels elevated in the CCl4/saline, and the spleen weight, QTc interval, and MDA and ALT levels were reduced by Gap 26 treatment. The level of nuclear factor (erythroid-derived 2) factor 2 (Nrf2) decreased in the CCl4/saline. The Cx43 expression was downregulated in the CCl4/saline and upregulated with the Gap 26 treatment.

Conclusion

Gap 26 not only alleviated the chronotropic hyporesponsiveness and the severity of liver damage and upregulated the atrial Cx43 expression, but it also had an antioxidant effect on the heart.

## Introduction

Cirrhosis is a long-term liver condition characterized by the presence of fibrosis and the expansion of blood vessels in the internal organs leading to the development of portal hypertension, which may lead to increased heart output, blood volume, and heart rate and decreased systemic vascular resistance [1,2]. Cirrhotic cardiomyopathy (CCM) is characterized by reduced heart reactivity, an elongated QT interval, and decreased systolic and diastolic functioning. Furthermore, inflammation and oxidative stress have roles in the development of cardiac abnormalities in patients with cirrhosis [3].

Connexins are proteins located across cell membranes and have crucial roles in the heart's functioning. Adenosine triphosphate (ATP), prostaglandin E2 (PGE2), glutamate, aspartate, and ions can be discharged from cells by the activation of hemichannels (HCs) [4,5]. Gap junctions (GJs) are crucial for facilitating intercellular communication. This enables the intercellular transport of molecules less than 1,000 daltons [6]. Gap junctional channels connect between the cytoplasm of adjacent cardiomyocytes, enabling the transmission of electrical signals. The predominant isoform in cardiomyocytes is connexin 43 (Cx43). Myocardial disorders such as hypertrophic cardiomyopathy, heart failure, and ischemia are associated with alterations in the expression and distribution of Cx43. Cx43 is found in the inner membrane of mitochondria implying its involvement in ischemia [7]. The expression and localization of Cx43 in the patient's heart exhibit alterations resulting in a reduction of typically four Cx43 molecules and their unequal redistribution towards the periphery of cardiomyocytes. Restoring Cx43 expression can reverse cardiac dysfunction and stabilize electrical activity. Cardiac remodeling is regulated by alterations in the structure and electrical activity, namely, through the aberrant expression of Cx43-based GJs which leads to a disruption in electrical coupling. The upregulation of Cx43 in liver tissues is concurrent with the occurrence of pathological damages [8]. Gap 26 is a peptide that mimics connexin and is composed of residues 63-75 of EL 1 of Cx43 and functions as an inhibitor of GJs. Gap 26 blocked the electrical connection between cells facilitated by GJs. Connexin mimetic peptides independently regulate HCs and GJs [9]. During the inflammatory process, GJs undergo rapid changes due to the limited lifespan of connexin [6]. Inflammation can lead to an upregulation of Cx HCs leading to a release of damage signals that perpetuate the inflammatory response [10]. The presence of Cx43 channels controls reactive oxygen species (ROS) inside the adjacent cells. When the function of the Cx43 channel was suppressed using Gap 26, the 15-F2t-isoprostane (a specific indicator of ROS) and lipid peroxidation products like malondialdehyde (MDA) and H2O2 were decreased, accompanied by an increase in the antioxidant enzyme superoxide dismutase (SOD). The presence of Gap 26 [11] reduced inflammatory factors such as interleukin-1 beta (IL-1β), interleukin-6 (IL-6), interleukin-8 (IL-8), and tumor necrosis factor-alpha (TNF-α). Gap 26 protects a healthy heart against ischemia-reperfusion injury [12]. This study aimed to assess the impacts of Gap 26 on chronotropic hyporesponsiveness, cardiac stress, inflammation, and oxidative stress in CCM. Additionally, it was investigated whether these possible effects were mediated by alterations in Cx43 expression.

## Materials and methods

Chemicals

All compounds used in the experiment were obtained from Sigma-Aldrich (UK).

Experimental animals

The experimental animal consisted of male Wistar albino rats (*Rattus norvegicus*) weighing between 200 and 230 g. These rats were prepared from the animal center of the Department of Pharmacology, Tehran University of Medical Sciences, Tehran, Iran. The experimental rats were raised by providing unlimited access to water and food consisting of rodent chow. The rats were subjected to a light/dark cycle of 12 hours each and were kept at a constant temperature of 22°C during the whole experiment. A sample of 60 rats was randomly chosen and divided into two groups: sham (n=24) and cirrhotic (CCl4-treated) (n=36) rats. The sham rats were categorized into three subgroups: sham/saline, sham/Gap, and cirrhotic (cir). Additionally, the group of rats with cirrhosis was further divided into subgroups: cir/saline, cir/Gap, and cir/sol. Among the groups, 12 rats were selected, with six rats being specifically utilized for in vivo examination of the QT interval, as the QTc interval, and for the assessment of chronotropic activity. The surviving rats (n=6) were utilized to test blood brain natriuretic peptide (BNP), aspartate aminotransferase (AST), alanine transaminase (ALT), heart TNF-α, nuclear factor (erythroid-derived 2) factor 2 (Nrf2), and MDA levels. Additionally, histological examination and investigation of atrial Cx43 gene expression were conducted.

Induction of cirrhosis

Liver cirrhosis was generated in rats by administering an intraperitoneal (IP) injection of 0.4 g/kg of CCl4. An emulsion with a ratio of carbon tetrachloride (CCl4), one part, to corn oil, six parts, was prepared and administered via injection three times per week for eight weeks. The ensuing investigations were conducted on the 56th day of CCl4 administration in the cirrhotic group or saline administration in the sham group [10].

Treatment protocol

The sham (saline) and cir (saline) groups received saline orally (PO) for eight weeks. Meanwhile, the sham (Gap) and cir (Gap) groups were given the Gap 26 solution (provided by the MedChemExpress company, code HY-P1082A) intraperitoneally in a mixture of dimethyl sulfoxide (DMSO) and corn oil at a dosage of 1 µg/kg/day. This treatment was administered for eight weeks, three times a week, half an hour before CCl4 administration. Since Gap 26 effectively blocks the electrical connection between cells through GJs after being applied for 30 minutes [9], we decided to administer Gap 30 minutes before the administration of CCl4. The cir (solvent) group received treatment using the solvent Gap 26, which consisted of a mixture of DMSO and corn oil, in equal proportion. The treatment was administered following the same therapeutic regimen as the Gap 26 treatment. Ultimately, sequential investigations were conducted upon completion of the therapy.

Histopathological staining

The heart tissue specimens, 2-3 mm in size, were fixed in a 10% formaldehyde solution of phosphate-buffered saline (PBS) for 24 hours. They were then transferred to 70% isopropyl alcohol for 180 minutes, followed by sequential immersion in isopropyl alcohol of increasing concentrations of 80%, 90%, and 100%, two hours each. Subsequently, the specimens were submerged in acetone (60-90 minutes) and introduced into xylene. The desiccated samples were immersed in liquefied paraffin wax, 58-60°C temperature range, within a blocked fallopian tube and then dried. Using a microtome, the samples were sectioned to 2-8 μm and transferred onto slides coated with Mayer's albumin consisting of egg white with glycerin (in equal amounts) with 1% sodium salicylate. This process was carried out at 60°C for two hours. After, xylene was used to remove paraffin from the tissues, for 20-30 minutes. Subsequently, a series of decreasing concentrations of isopropyl alcohol was employed to rehydrate the tissues, with each concentration being applied for about 2-3 minutes. The tissues were immersed in water for three minutes, subsequently transferred to hematoxylin for about 1-2 minutes, and finally rinsed with tap water for about 1-2 minutes. Subsequently, the tissues that had been mounted were transferred to hydrochloric acid (HCl) for one minute, followed by immersion in distilled water (a solution containing 20 g magnesium sulfate, 3.5 g sodium carbonate, and 1 L distilled water) for one minute. Finally, the tissues were stained with eosin dye for 30 seconds. The isopropyl alcohol was employed increasingly to remove moisture from the tissues, which were immersed in xylene for 20-30 minutes. Subsequently, a single droplet of dibutylphthalate polystyrene xylene mounting agent was applied to the slides, which were then left to dry for the duration of the night. Later, the slides were scrutinized using the Olympus BX51 light microscope from Japan, at a magnification of 100×, and were documented [12]. The histological alterations observed in CCM encompass fibrosis, subendocardial edema, as well as vacuolation of the nucleus and cytoplasm in myocardial cells. Nevertheless, these findings lack specificity and are challenging to visualize with a light microscope.

Electrocardiography (ECG)

The ECG was performed on the cirrhotic rats after administering anesthesia via IP injection of ketamine (100 mg/kg body weight) and diazepam (0.4 mg/kg body weight) with appropriate dosage modification for diazepam. Three stainless-steel electrodes were inserted under the skin to measure the lead II electrocardiogram, and the ECG was recorded for five minutes. The ECG was linked to a bio-amplifier to enhance the signals (the PowerLab system, ADInstruments, Australia). The PowerLab system utilized its analog or digital converter to transform the amplified sounds into digital format at a frequency of 10 kHz. The signals were observed and documented using LabChart 7 software from ADInstruments. The QT interval data were presented as the QTc interval, calculated using Bazett's formula as follows: QTc=QT/√R-R [13].

In vitro chronotropic study

A chronotropic research was conducted on the atria obtained from sedated rats. The spontaneously beating atria were extracted from the isolated hearts and subjected to a chronotropic investigation following stimulation with isoproterenol at several doses (ranging from 10-10 to 10-5 M). The atria were placed in a cold physiological salt solution (PSS) that was supplied with a high concentration of carbogen gas (95% O2 and 5% CO2). The atria which had been separated from other tissues were thereafter placed in the organ bath with 20 mL of physiological PSS and subjected to an isometric force of 1 g. The heat and pH of the bath were controlled at a constant level of 37.0±1°C and 7.4. The composition of the PSS was in millimolar (mM) [14]. The carbogen gas was applied to oxygenate the PSS within the organ bath. In the end, the PowerLab equipment monitored the atrial beating rates specifically in the right atrium which contains the sinoatrial node (SA node) [14]. Different isoproterenol concentrations ranging from 10-10 to 10-5 M were added to stimulate the atrial beating responses [13].

Serum BNP, ATL, AST, and heart TNF-α and Nrf2 levels

Enzyme-linked immunosorbent assay (ELISA) was carried out to calculate serum BNP and heart tissue TNF-α and Nrf2 levels in the sham and cirrhotic rats. For BNP, AST, and ALT measurements, the blood was taken directly from the heart, and the serum was collected. The tubes then were kept at room temperature for a while. To measure TNF-α and Nrf2, the tissue samples were taken from the isolated hearts in PSS supplied with a carbogen gas. The samples were preserved in liquid nitrogen, and they were deep frozen at -120°C temperature for other assessments. The tissues were homogenized in PBS and were centrifuged at 1000 g for 15 minutes for TNF-α and 20 minutes for Nrf2, and the supernatant was collected. The ELISA was carried out using the Rat BNP ELISA kit of a 96-well plate "Cat. No. MBS764880, MyBioSource, Inc., San Diego, USA" [14,15], Rat TNF-α ELISA kit of a 96-well plate "Cat. No. SK00109-02, Aviscera Bioscience, Inc., Santa Clara, CA" [14,16], and Rat Nrf2 ELISA kit of a 96-well plate "Cat. No: MBS012148, MyBioSource, Inc., San Diego, USA" [14] according to the manufacturer's instructions. Isoproterenol (10-10 to 10-5 M) was added to stimulate the atrial beating responses [13].

Heart MDA level measurement

The current investigation involved the collection and preservation of cardiac samples to measure the amount of MDA in the heart. The TCA-TBA-HCl chemical reagent was made by swirling 15% w/v TCA, 0.375% w/v TBA, and O.25 N HCl and then slightly heating the mixture dissolving the TBA. Subsequently, the 2.0 mL reagent was added to a 1 mL portion of a 2 mg heart sample and was subjected to boiling for 15 minutes in a water bath. Following the cooling of the solution, centrifugation was performed at a speed of 10,1000 g for 10 minutes to remove the fluffy precipitate. Ultimately, the spectrophotometer (Unico, 2100 Spectrophotometer, New Jersey, USA) was used to measure the absorbance of both the samples and blanks. The blanks consisted of all the reagents except for the lipid. The absorbance readings were taken at a wavelength of 535 nm. The MDA content in the sample was determined using an extinction coefficient of 1.56×105 M -1 cm1 [14,17].

Real-time polymerase chain reaction (RT-PCR): atrial Cx43 expression

Quantification of atrial Cx43 mRNA expression levels was done using RT-PCR. Atrial Cx43 mRNA expressions were quantified using RT-PCR. The atria were excised from the isolated hearts and placed in "PSS with a carbogen gas supply. After that, the atrial samples were stored. The total RNA extraction was performed using the RNeasy fibrous tissue mini kit (Qiagen, Germany). The DNase (Promega) was employed to eradicate deoxyribonucleic acid (DNA). The RNA sample (10 µL) was formed by combining the reaction buffer (10 µL) with RNA which was treated with deoxyribonuclease (1 µL). The combination was exposed to a temperature of 70°C for five minutes. After that, it was transferred to ice packs to chill. The following components were combined in a bottle: reverse transcriptase (1 µL), random hexamer primer (d (N) 6) (0.5 µL), oligo dT20 primer (0.5 µL), deoxynucleoside triphosphates (1 µL), ribonuclease inhibitor (1 µL), and reaction buffer (10 µL). At that moment, the mixture was incubated, at 42°C for one hour, to synthesize the first strand of complementary DNA (cDNA). The oligonucleotide primers used for amplifying the rat atrial Cx43 and glyceraldehyde 3-phosphate dehydrogenase (GAPDH) genes were as follows: Connexin 43 [Forward: 5’-GCCTCCAAGGAGTTCCACCA-3’, Reverse: 5’- GTGGAGTAGGCTTGGACCTT-3’] GAPDH [Forward: 5’-GGC ACA GTC AAG GCT GAG AAT G-3’, Reverse 5’-ATG GTG GTG AAG ACG CCA GTA-3’] [14]. The RT-PCR was carried out by combining 10 pmol of the forward and reverse oligonucleotide primers, 1 µL of cDNA template, and 10 µL of optimized PCR master mix, generating a final volume of 20 µL. The reactants' combination was processed using a Rotor-Gene machine. The RT-PCR data was normalized using an internal reference gene, GAPDH. The thermal cycler methodology consisted of an initial denaturation step at 95°C for five minutes, followed by annealing at 60°C for 45 seconds (repeated for 45 cycles), and extension/elongation at 72°C for 30 seconds [13]. The RT-PCR data were represented by the cycle threshold (CT) value which indicated the cycle number required for the fluorescence signals to surpass a specific threshold. The differences in CT values between the targeted cDNA and internal control genes were denoted as ∆CT. A decrease in ∆CT value indicated a higher mRNA level." The RT-PCR efficiency was similar for both the targeted and internal control genes, thus allowing for relative quantification using ∆CT [14,18].

Statistical analysis

Statistical analysis was conducted on the collected data using various methods. The mean±SEM was used to present the data. ANOVA compared the treatments in the sham and cirrhotic groups. Two-way ANOVA was used for the analysis of chronotropic data, and post hoc Bonferroni's test was used for multiple comparisons. One-way ANOVA analyzed the QTc interval, BNP, ALT, AST, TNF-α, Nrf2, MDA, and atrial Cx43 mRNAs. Post hoc Tukey's test was used to determine significant differences. Statistical significance was considered when P<0.05. GraphPad Prism 5.0 (Inc., San Diego, CA, USA) was used for data analysis.

## Results

On the 56th day, all rats treated with CCl4 exhibited jaundice symptoms, including increased abdominal fluid (ascites) and yellow urine, in contrast to the sham animals. We visually assessed the liver stiffness and quantified spleen weight in all experimental animals to establish the presence of cirrhosis. Animals treated with CCl4 had a significantly increased spleen weight (1.11±0.11 g) compared to the sham group (0.86±0.12 g) with a P-value of 0.0004. Rats treated with CCl4 exhibited elevated hepatic stiffness compared to the control group which were indicative of portal hypertension.

Spleen weight/body weight changes

**Figure 1:**
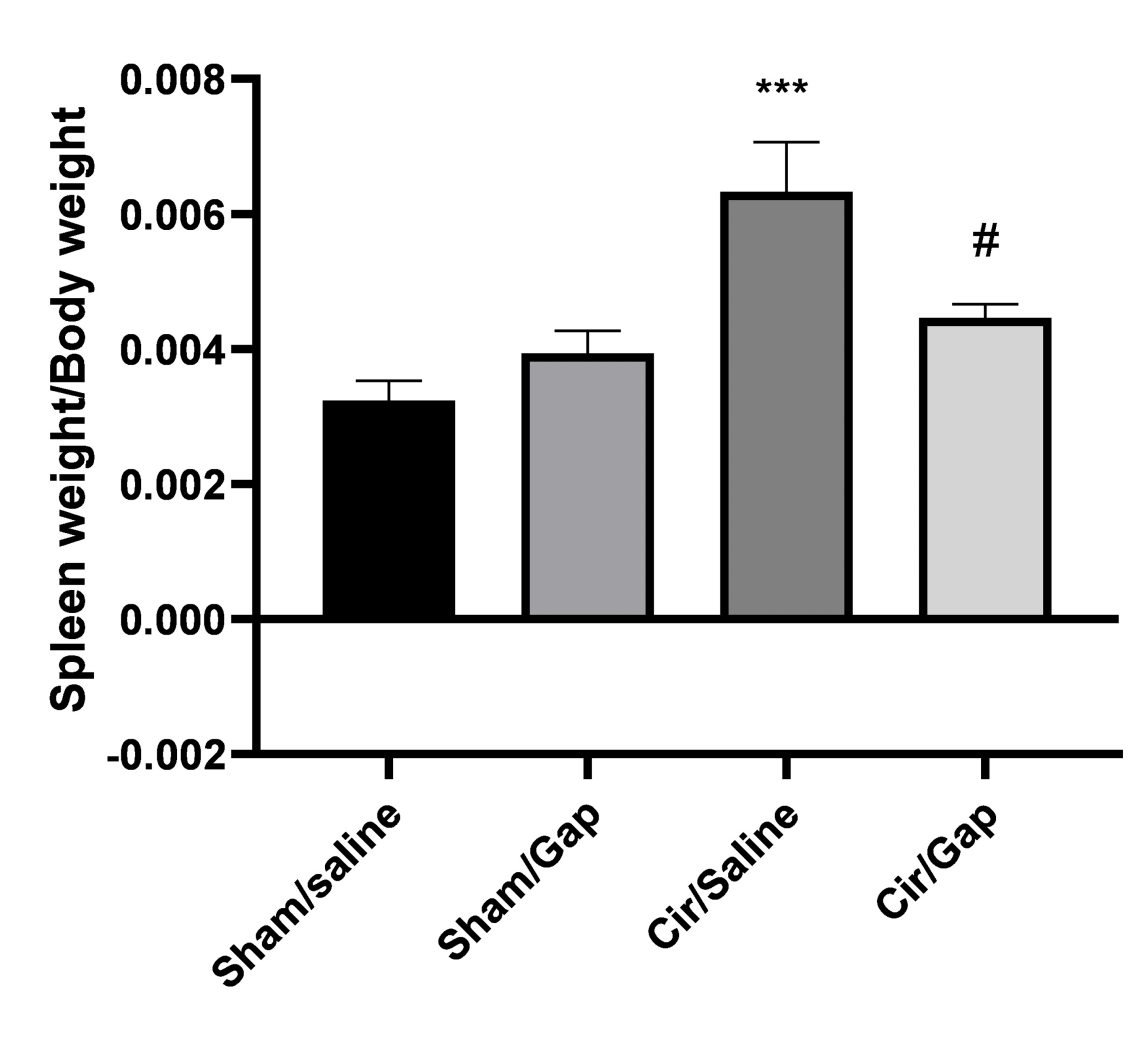
The spleen weight/body weight changes of sham (control) or CCl4-treated rats following saline or Gap 26 (1 µg/kg, PO) treatment. One-way ANOVA followed with post hoc Tukey's test, and the outcome was considered significant at P<0.05. The data are presented in mean±SEM. Six to eight rats were included in each experimental group. ***P<0.001 compared to the sham/saline group. #P<0.05 compared to the cir/saline group. Cir: cirrhotic; sol: solvent

The spleen weight/body weight changes were calculated for both sham and CCl4-treated groups at the end of the treatment. Compared to the sham/saline group, the sham/Gap 26 groups represented no significant change (P>0.05). The cir/saline group revealed a significant increase (P<0.001) compared to the sham/saline group. The cir/Gap 26 group showed a notable decrease (P<0.05), in comparison to the cir/saline group. Figure 1 displays the spleen weight/body weight statistics for both the sham and cirrhotic groups.

Histopathological study

**Figure 2:**
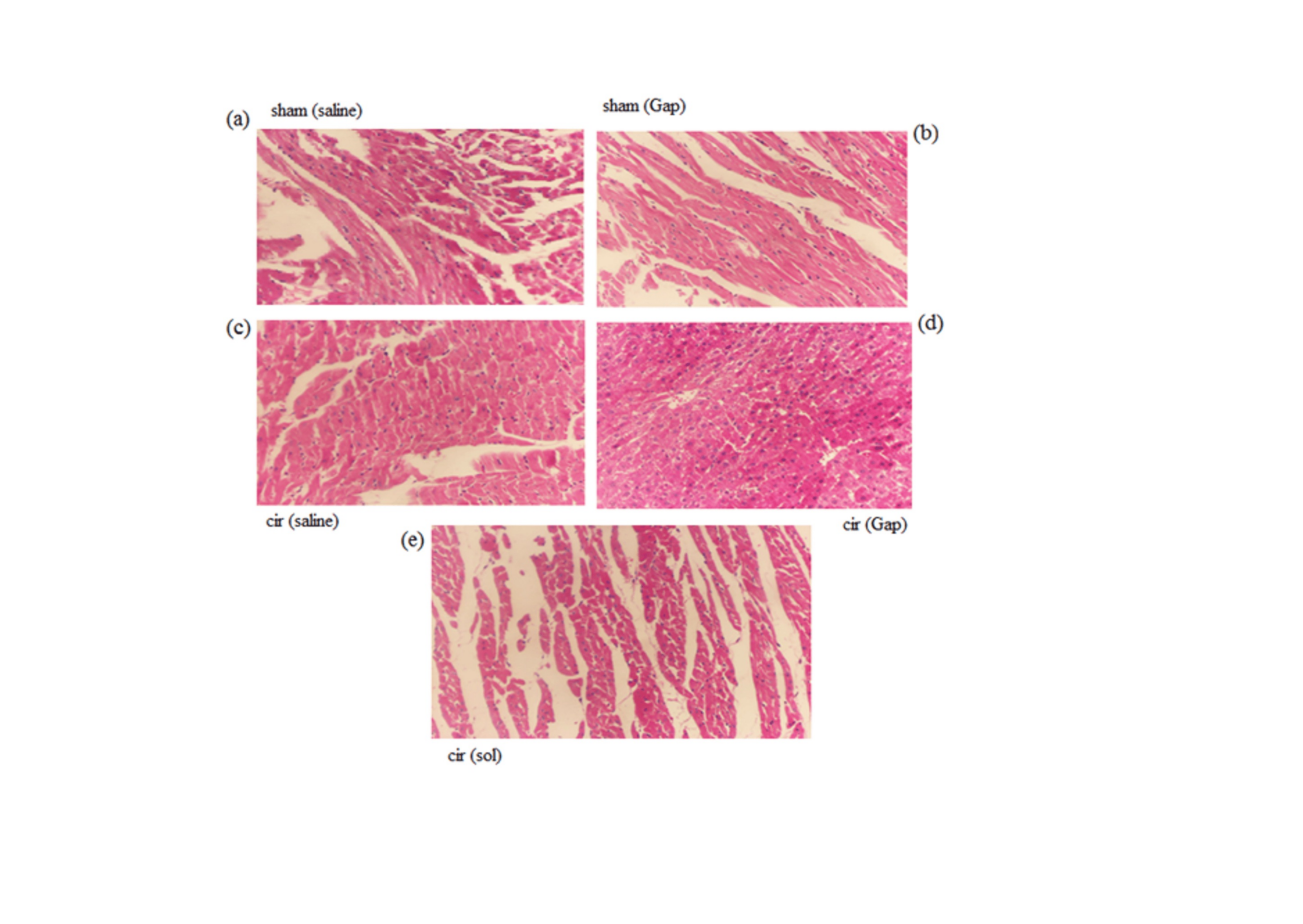
The heart sections' histopathology photomicrographs of sham (control) (Figure 2a) and CCl4-treated (cirrhotic) rats (Figure 2b) after saline treatment (Figure 2c) or Gap 26 (1 µg/kg, PO) (Figure 2d). The photomicrographs (100×) are shown in 56 days of treatment. The H&E was used for staining heart sections. Six rats were included in each experimental group. Cir: cirrhotic; sol: solvent

We evaluated the histopathology of the left ventricles with a light microscope. Histopathological examination of a rat's heart (H&E×100) is shown in Figure 2. Histologic changes of myocardial cells in CCM include fibrosis, subendocardial edema, vacuolation of the nucleus and cytoplasm, fibrosis, and inflammation [18]. The histopathology study showed mild inflammation in only one rat in the sham/saline group. No abnormal symptoms were seen in the sham/Gap group. In the cir/saline group, we did not see any criteria for cirrhotic histopathology. In the cir/Gap group, mild inflammation was seen in one of the rats. The inflammation was seen in two animals in the cir/Gap group. In the cir/sol group, we saw mild inflammation only in one animal.

QT interval analysis

**Figure 3:**
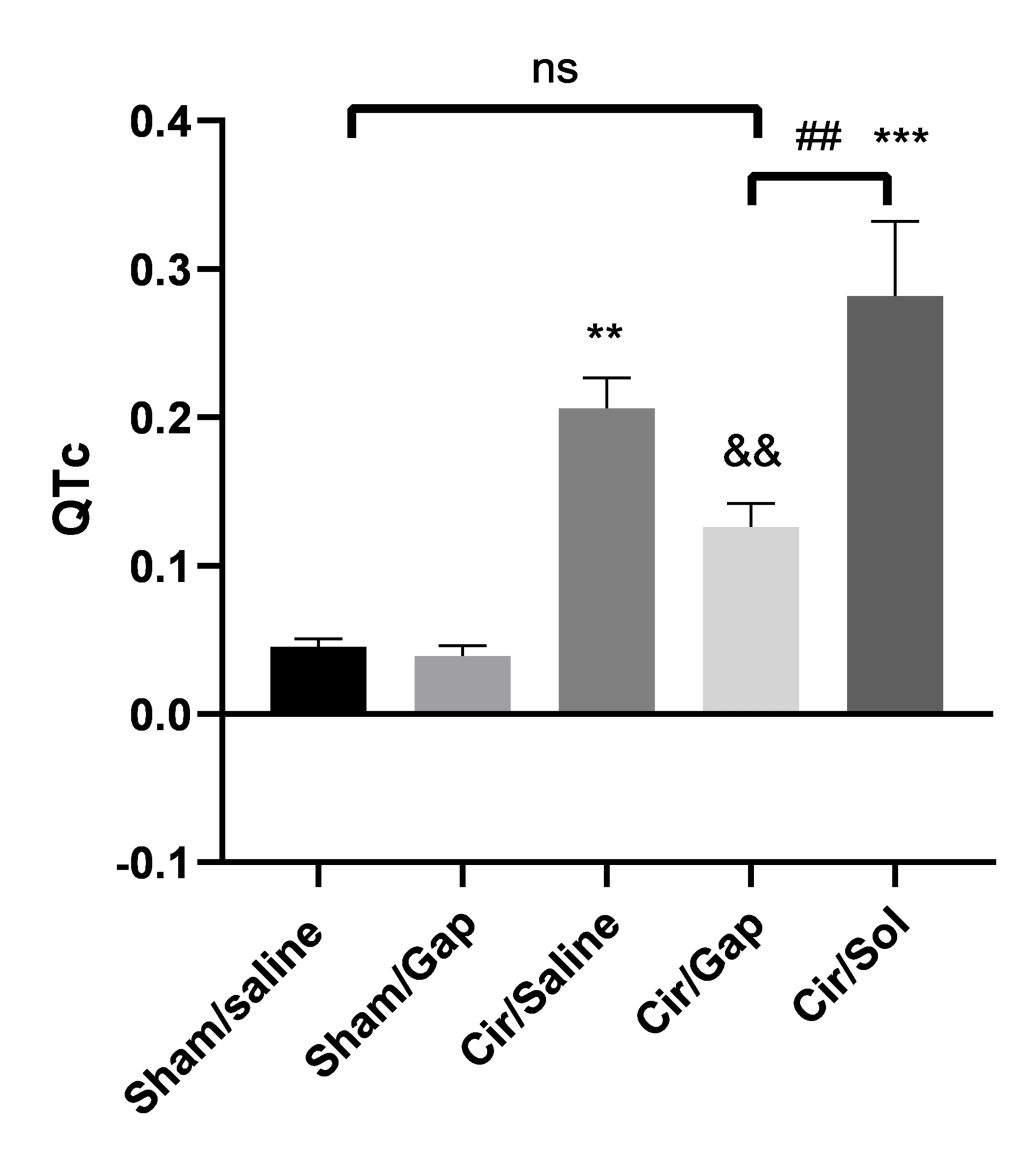
The QTc intervals of sham (control) or CCl4-treated rats (0.4 g/kg, IP) after treatment with saline or Gap 26 (1 µg/kg, PO). The data for respective groups are mentioned after 56 days of treatment. One-way ANOVA was applied to analyze data with post hoc Tukey's test, and the significance level was P<0.05. The outcome is shown as mean±SEM, n=6. **P<0.01, ***P<0.001 compared to the sham/saline group. &&P<0.01 compared to the cir/saline group. ##P<0.01. Cir: cirrhotic; sol: solvent

The QT intervals, as corrected QT (QTc), were measured for the sham and CCl4-treated groups and are given in Figure 3. In comparison to the sham/saline group, the sham/Gap showed no significant change (P>0.05) in the QTc interval. Nevertheless, the cir/saline and cir/sol groups revealed marked increases (P<0.01 and P<0.001, respectively) in the QTc interval compared to the sham/saline group. In comparison to the cir/saline and cir/sol groups, the cir/Gap group represented a substantial decrease (P<0.01) in the QTc interval.

Average atrial beating rate

**Figure 4:**
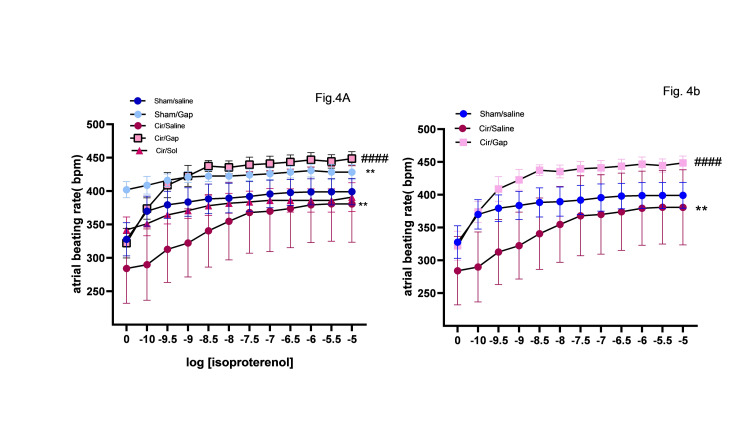
Average atrial beating rates of sham (control) and CCl4-treated (0.4 g/kg, IP) (cirrhotic) rats after treatment with saline or Gap 26 (1 µg/kg). The atrial beating rates were calculated pre- (4B) and post-stimulation with 10-10 to 10-5 M cumulative isoproterenol (4A). The data for respective groups are mentioned after 56 days of treatment. Two-way ANOVA was applied to analyze data with post hoc Bonferroni's test, and the results are significant at P<0.05. The data are presented as mean±SEM and n=6 rats. **P<0.01 compared to the sham/saline group. ####P<0.0001 compared to the cir/saline group. Cir: cirrhotic; sol: solvent

The average atrial beating rates were calculated for sham and CCl4-treated groups after treatment with saline or Gap 26, as shown in Figure 4. The atrial beating rates were assessed in vitro following the various isoproterenol concentrations, i.e., 10-10 to 10-5 M. Our data showed that chronotropic responses to isoproterenol were impaired significantly in isolated atria following chronic treatment with CCl4 (F CCl4 vs. sham=3.158, P<0.001, Figure 4A). Also, there was a significant difference in EC50 of isoproterenol between the CCl4 group (log EC50=8.78) and the sham-operated groups (log EC50=10.1) (P<0.01). As shown in Figure 4A, Gap 26 could increase the chronotropic responsiveness of isoproterenol in the sham-operated group (F saline vs. gap=130.7, P<0.01). In Figure 4A, 4B, the log EC50 of isoproterenol changed significantly in the cir/saline group (log EC50=8.78), as compared to the CCl4/Gap group (log EC50=9.83) (P<0.01).

Blood BNP, AST, and ALT levels

**Figure 5:**
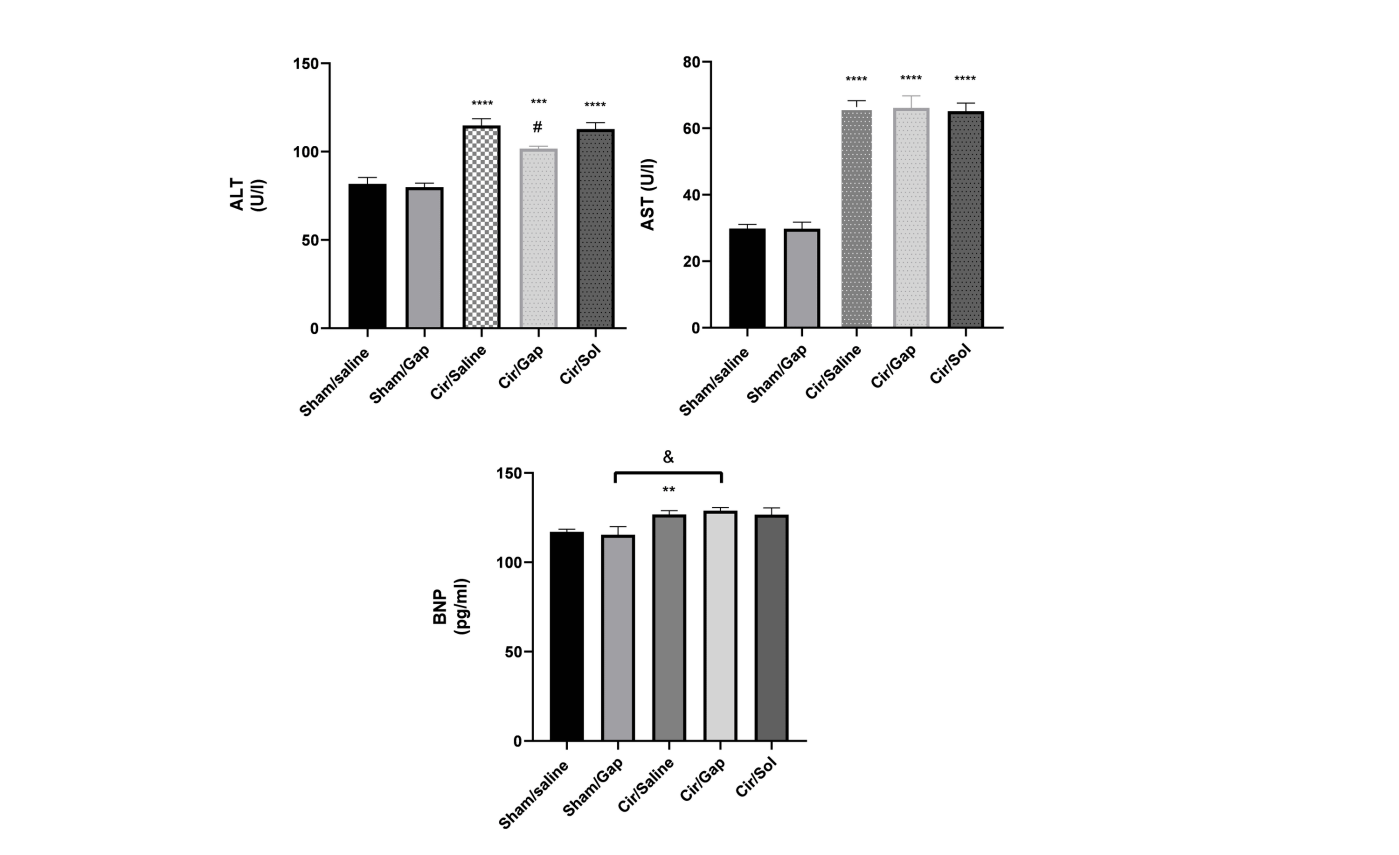
The serum BNP, ALT, and AST levels of sham (control) and CCl4-treated rats (0.4 g/kg, IP), after treatment with saline or Gap 26 (1 µg/kg). The data are mentioned 56 days post-treatment. One-way ANOVA with post hoc Tukey's test analyzed the data, and the significant difference is at P<0.05. The data are presented as mean±SEM. Six rats were included per experimental group. **P<0.01, ***P<0.001, ****P<0.0001 compared to the sham/saline group. &P<0.05 compared to the sham/saline group. #P<0.05 compared to the cir/saline group. Cir: cirrhotic; sol: solvent; BNP: brain natriuretic peptide; ALT: alanine transaminase; AST: aspartate aminotransferase

There was a significant difference between sham/Gap and CCl4/Gap groups, and the only variable between them is the chronic treatment with CCl4. Thus, it can be said that CCl4-induced cirrhosis has been able to increase the BNP level significantly. In comparison to the sham/saline group, the sham/Gap group did not exhibit a statistically significant alteration in the serum BNP level. The serum BNP level did not show a significant difference (P>0.05) in the cir/Gap group compared to the cir/saline group. Therefore, Gap 26 administration did not impact the BNP level in the CCL4-treated group (Figure 5a). Figure 5b shows a substantial rise (P<0.0001) in serum ALT levels in the CCl4/saline group compared to the sham/saline group. The serum ALT levels in the sham/Gap group did not show a significant difference compared to the sham/saline group (P>0.05). The cir/Gap group showed a substantial decrease compared to the cir/saline group (P<0.05). Serum AST levels were evaluated for both the sham and CCl4 groups. The CCl4/saline group showed a statistically significant increase (P<0.0001) in serum AST level compared to the sham/saline group. The serum AST levels in the sham/Gap group did not show a statistically significant difference compared to the sham/saline group (P>0.05). The CCl4/Gap group did not show a statistically significant difference compared to the CCl4/saline group (P>0.05).

Cardiac TNF-α, Nrf2, and MDA levels

**Figure 6:**
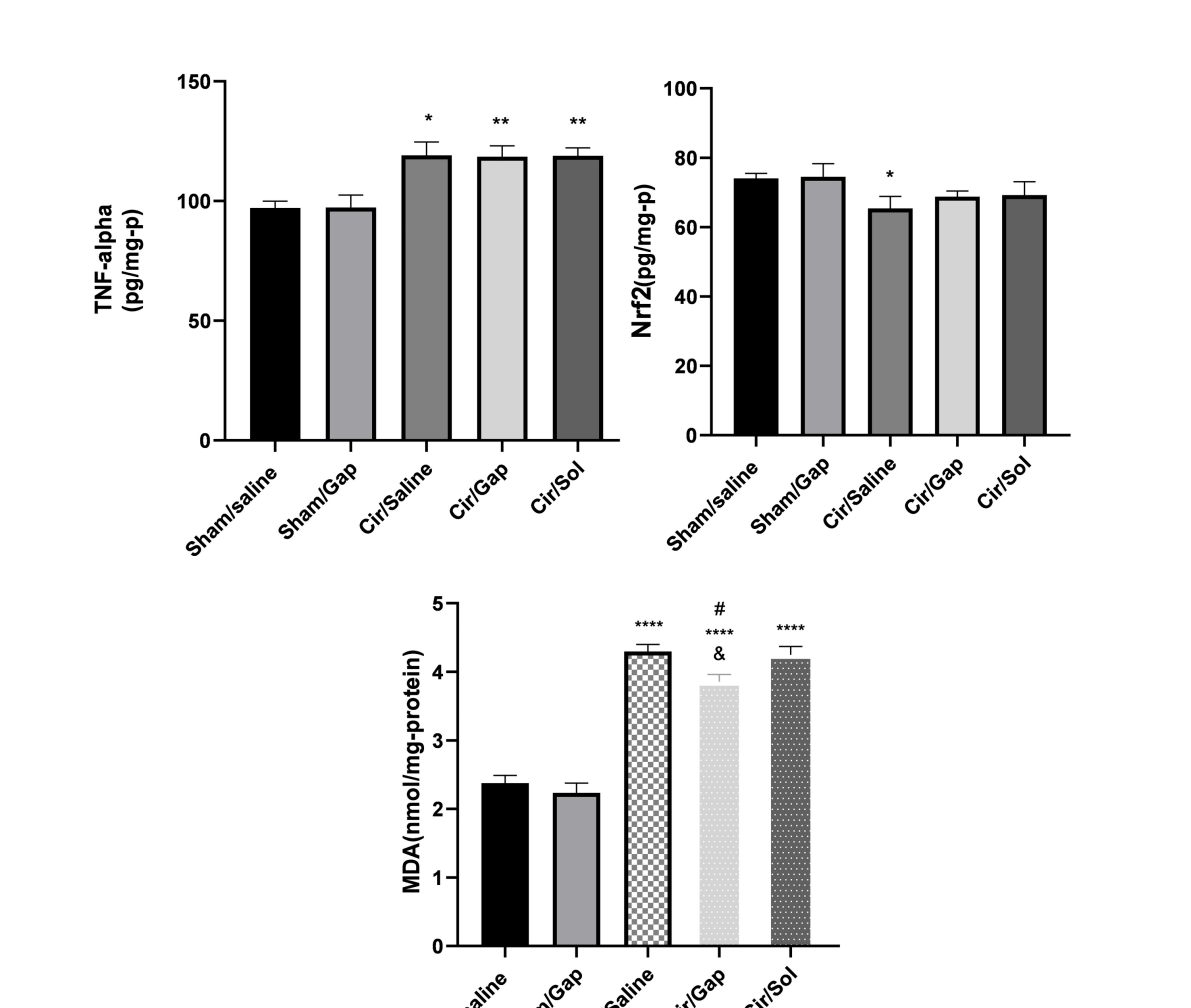
The tissue levels of TNF-α, Nrf2, and MDA in sham (control) and CCl4-treated (0.4 g/kg, IP) rats after treatment with saline or Gap 26 (1 µg/kg, PO). The data for the respective groups are mentioned after 56 days of treatment. One-way ANOVA was applied to analyze data with post hoc Tukey's test, and the significant level was at P<0.05. The data are presented as mean±SEM. Six rats were included per experimental group. *P<0.05, **P<0.01, ****P<0.0001 compared to the sham/saline group. #P<0.05 compared to the cir/saline group. &P<0.05 compared to the cir/sol group. Cir: cirrhotic; sol: solvent; TNF-α: tumor necrosis factor-alpha; Nrf2: nuclear factor (erythroid-derived 2) factor 2; MDA: malondialdehyde

Figure 6 displays the levels of cardiac TNF-α, Nrf2, and MDA in both the sham and CCl4 groups. The CCl4/saline group exhibited a notable rise in cardiac TNF-α levels compared to the sham/saline group (P<0.05), as seen in Figure 6a. There was no significant difference (P>0.05) in the cardiac TNF-α level between the sham/Gap group and the sham/saline group. No significant difference (P>0.05) was observed in the cardiac TNF-α level between the CCl4/Gap group and the CCl4/saline group (Figure 6a). The Nrf2 levels in the heart were evaluated for both the sham and cirrhotic groups, as shown in Figure 6b. The findings did not reveal any statistically significant difference (P>0.05) between the sham/Gap group and the sham/saline group. The CCl4/saline group showed a statistically significant difference compared to the sham/saline group (P<0.05). The Nrf2 level in the heart of the CCl4/Gap group did not show a statistically significant change (P>0.05) when compared to the CCl4/saline group (Figure 6b). The myocardial MDA levels were assessed for both the sham and cirrhotic groups, as illustrated in Figure 6c. The CCl4/saline group showed a statistically significant increase compared to the sham/saline group (P<0.0001). The cardiac MDA levels in the sham/Gap group did not show a significant difference compared to the sham/saline group (P>0.05). The CCl4/Gap group showed a statistically significant difference compared to the CCl4/saline and CCl4/sol groups (P<0.05) (Figure 6c).

Expression levels of atrial Cx43 gene mRNAs

**Figure 7:**
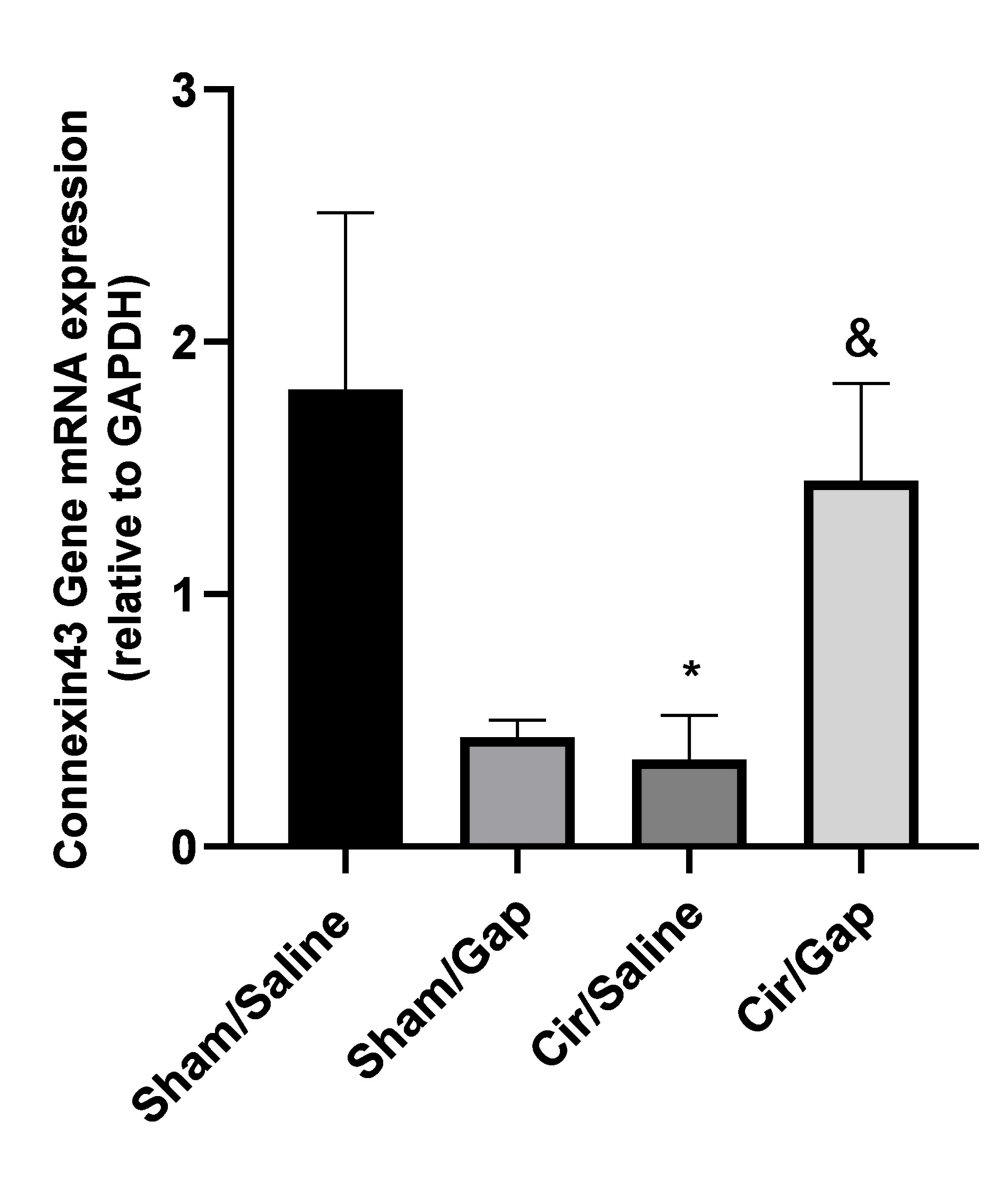
The tissue levels of TNF-α, Nrf2, and MDA in sham (control) and CCl4-treated (0.4 g/kg, IP) rats after treatment with saline or Gap 26 (1 µg/kg, PO). The data for the respective groups are mentioned after 56 days of treatment. One-way ANOVA was applied to analyze data with post hoc Tukey's test, and the significant level was at P<0.05. The data are presented as mean±SEM. Six rats were included per experimental group. *P<0.05, **P<0.01, ****P<0.0001 compared to the sham/saline group. #P<0.05 compared to the cir/saline group. &P<0.05 compared to the cir/sol group. Cir: cirrhotic; sol: solvent; TNF-α: tumor necrosis factor-alpha; Nrf2: nuclear factor (erythroid-derived 2) factor 2; MDA: malondialdehyde

The expression levels of atrial Cx43 gene mRNAs were quantified for the sham and CCl4-treated groups as given in Figure 7. A non-significant decline (P=0.06) was noticed in the atrial Cx43 mRNA level of the sham/Gap group compared to the sham/saline group. Compared to the sham/saline group, the CCl4/saline group showed a significant drop (P<0.05). In addition, the CCl4/Gap group revealed a significant increase (P<0.05) in comparison to the CCl4/saline group.

## Discussion

We evaluated the effects of Gap 26, a specific Cx43-inhibiting peptide, on CCl4-induced cardiomyopathy in rats. In cirrhotic rats, ECG showed prolonged QTc interval. Moreover, the spleen weight, serum BNP, ALT, AST, and cardiac TNF-α and MDA increased, while tissue Nrf2 decreased. In this way, arterial Cx43 gene expression was reduced. On the other hand, we found for the first time that chronic treatment with Gap 26 may improve some of the cardiac complications resulting from cirrhosis. Accordingly, Gap 26 increased the chronotropic hyporesponsiveness to β-agonist, improved the QTc abnormality, and increased the atrial Cx43 gene expression. Besides that, it decreased the spleen weight and oxidative stress markers MDA and ALT. Nonetheless, Gap 26 had no effects on heart histopathology, the cardiac stress marker BNP, the cardiac inflammation marker TNF-α, and the antioxidant activity Nrf2. Long-term CCl4 treatment induced cirrhosis and increased spleen index due to portal hypertension [19]. In line with this, in our experiment, the spleen weight increased in cirrhotic animals. In addition, in CCl4-induced cirrhosis and alcoholic and post-viral hepatitis, the QTc interval is prolonged [20]. In some chronic cholestatic liver diseases, the CCM is directly associated with cardiac hypertrophy resulting from bile acids, cardiomyocyte apoptosis, prolongation of QT interval, and irregular hemodynamics [14,21]. These studies are somewhat similar to our findings. Furthermore, we found that CCl4 increased serum ALT and AST. In a study, the serum levels of ALT, AST, and ALP in the CCl4-treated group increased, demonstrating that liver cirrhosis is successfully established [22] which supports our finding. 

On the other hand, our study demonstrated that the Gap 26 treatment decreased the QTc interval in cirrhotic rats. The presence of Cx43 in the mitochondria and Gap 26 conferred protection to an intact heart against myocardial ischemia injury [12]. After a myocardial infarction, Cx43 was found in the myocytes. Localization of Cx43 was considered important for anisotropic conduction in a normal heart, and this "lateralization" of Cx43 was suspected to be involved in alterations of conduction in the injured heart [23]. Alterations in Cx43 expression and distribution were observed in myocardial disease [24]. Cx43 has been linked to cardiac arrhythmia and diseases of the heart including ischemic heart disease and heart failure, and several Cx43 mimetic peptides have advanced to clinical testing. There is also evidence that repeated dosing of EL-based peptides can have beneficial models in chronic heart diseases [25]. The major GJ protein expressed in the heart, Cx43, is highly remodeled in the diseased heart. Usually, Cx43 is downregulated and heterogeneously redistributed to the lateral sides of cardiomyocytes. Reverse remodeling of the impaired Cx43 expression could restore normal cardiac function and normalize electrical stability [26]. We found that Gap 26 decreased the spleen weight in cirrhotic rats. The decrease of the spleen weight coincides with the decrease of the ALT enzyme in the Gap-treated group. This can be due to some degree of improvement in the liver. Our previous study found that when atorvastatin decreased the spleen weight, necroinflammation of the liver decreased and this coincided with improving the chronotropic responsiveness and decreasing the QTc interval, which is also consistent with the results of the current study [14]. In our study, the Cx43 inhibitor decreased ALT, but not AST. ALT and AST are increased in liver damage and are used to screen for and/or monitor liver disease. Conditions associated with moderately high levels of ALT and AST are chronic liver disease, heart damage, kidney damage, and muscle injury [27,28]. Because ALT is present primarily in the liver, it is a more specific marker of hepatocellular injury compared to AST which is present in the liver as well as other organs [29]. Thus, an increase in AST without an elevation in ALT, as we found in the Gap 26-treated cirrhotic group, is suggestive of other organ diseases. Therefore, it seems that Gap 26 prevents liver damage.

In our investigation, the chronotropic responses were decreased in cirrhotic animals. The major features of CCM are impaired beta-adrenoceptor function, plasma membrane dysfunction, and increased CO. Other potential mechanisms are the production of cardio-depressants such as endotoxins, endothelin, cytokine bile salts, and nitric oxide (NO) [30]. Our experimentation for the first time showed that Gap 26 may increase the chronotropic responses in cirrhotic hearts.

MDA and SOD concentrations raised cirrhosis in both the serum and the ascitic fluid [31]. The MDA concentration is associated with the severity of liver fibrosis in cirrhosis positively. We demonstrated that they are correlated [32] and that the heart MDA goes up in cirrhotic rats. Also, the MDA level is well-matched with cirrhosis. Moreover, we found that the Gap 26 treatment lowered the heart MDA level in cirrhosis. The serum level of MDA is significantly higher in patients with cirrhosis [33]. These studies show similarities to our findings. Selective inhibition of Cx43 HCs with Gap 19 protected human umbilical vein endothelial cells from lipopolysaccharide (LPS)-induced apoptosis since the increased production of ROS and apoptosis elicited by LPS and Cx43 overexpression were reduced with Gap 19 [34]. These studies show similarities to our findings. Also, treatment with a mitochondrially targeted antioxidant, MitoTEMPO, reduced sudden cardiac death in ACE8/8 mice; decreased spontaneous ventricular premature beats, ventricular tachycardia inducibility, and mitochondrial ROS; prevented structural damage to mitochondria; resulted in an increase in Cx43 at the GJs; and corrected GJ conduction [35]. In this study, the atrial Cx43 mRNA expression level dropped in cirrhotic hearts, and Gap 26 could increase atrial Cx43 mRNA expression. Congestive heart failure is associated with reduced principal GJ protein, Cx43, in the left ventricle, contributing to enhanced arrhythmogenicity and contractile dysfunction. This downregulation is due predominantly to a reduced transcript steady-state level [36].

CCl4 cirrhosis in rodents reproduces the main characteristics of human cirrhosis. The liver is grossly nodular, and there is portal hypertension in most of the animals. This model shares several features with alcoholic human cirrhosis [37]. BNP level increases in post-hepatitis C cirrhotic patients, and it is correlated with both the severity of the liver disease and the morphofunctional cardiac changes. The relationship between BNP level and myocardial function is complex and is altered by liver disease [38]. Serum BNP is correlated with hepatocellular failure and portal hypertension-lowering therapies, and then it can be used as a valuable parameter in monitoring the therapy response [39] which is not similar to our results. In the mentioned study, the patients were evaluated for 20 months, but the duration of our study was two months. Although in our experiment Gap 26 demonstrated protective effects, Gap 26 did not decrease BNP.

Cardiac histological changes have been described in several autopsy studies including myocardial hypertrophy, cardiomyocyte edema, fibrosis, nuclear vacuolation, and unusual pigmentation [17]. However, these changes were reported from studies dating back at least 50 years in patients with alcoholic cirrhosis. The autopsies of 108 patients from all causes (most alcoholic) demonstrated the same cellular myocardial abnormalities [40]. Some studies conducted on animal models on long-term light microscopy demonstrated histological changes in the heart tissue, for example, the presence of dilated and congestion vessels showed moderate lesions. Although the myocytes were normal, mononuclear infiltration, edema, and moderate inflammation were observed in most of the interstitium parts [41]. Notably, there are experiments in which no histological differences were reported. This discrepancy between the histological changes in human and animal studies is probably related to the long disease duration in cirrhotic patients versus the much shorter periods needed to induce cirrhosis in animal models [42]. Similarly, we did not find any changes in the histopathology of the heart.

In the current study, the levels of TNF-α were higher in the myocardial tissues of the experimental animal group. We demonstrated that CCl4-induced cirrhosis caused inflammation in the heart and Gap 26 could not prevent the inflammation. Besides, we found that Cx43 expression decreased in the CCl4-treated group. In one study, TNF-α directly inhibited Cx43 expression in a dose- and time-dependent manner in vascular smooth muscle cells [43]. In our study, the Nrf2 level was diminished in cirrhotic rats' hearts. The Nrf2 is stimulated using nitrogen-based and oxygen-derived radicals resulting from metabolic sources [16] and activates the expression of the multidrug resistance-associated proteins (MRPs), NAD(P)H quinone oxidoreductase 1 (NQO1), and heme oxygenase-1 (HO-1) genes which act against liver diseases in several ways [14]. Under stressful conditions, oxidative or electrophilic molecules react with the cysteine residues of Keap1 to cause conformational changes in it, leading to the degradation of Nrf2 [44]. This report supports our finding that Gap 26 could not increase Nrf2. Selective inhibition of Cx43 HCs reported to protect umbilical vein endothelial cells against LPS stimulation via a reduction in ROS [34]. Gap 26 confers protection against ischemia-reperfusion injury to the intact heart via the inhibition of Cx43 HCs [12]. Oxidative stress increased Cx43 expression and Cx43 GJ-mediated intercellular communication. Cx43 GJ-mediated intercellular communication amplified oxidative stress signaling in the lungs, inducing excessive apoptosis via the ASK1-JNK/p38 signaling pathway [44].

In this research, for the first time, the medicinal route of closing connexins has been used to prevent the transfer of toxic substances between the heart and liver cells, which has had promising results. Also, the effects of peptide drugs on CCM have been investigated. In the next studies, it is necessary to check the level of Cx43 protein expression, which is one of the shortcomings of our study. Moreover, this study in inotropic heart models and complete heart models can determine the effect of the drug on the strength of heart contraction and the interaction of this drug with other organs in CCM.

## Conclusions

In brief, Gap 26 relieved the chronotropic hyporesponsiveness, decreased EC50, increased maximum response to it, and improved the QTc abnormality in cirrhotic rats. However, it did not affect the heart histopathology. Gap 26 could not reduce the cardio marker of stress, inflammation, and antioxidant activity, but did decrease oxidative stress and ALT. Gap 26 increased the atrial Cx43 gene expression in cirrhotic hearts. The use of Gap 26 might be effective in CCM through its antioxidant and anti-inflammatory effects and, also, the upregulation of Cx43.
