# Correction: Effects of Gap 26, a Connexin 43 Inhibitor, on Cirrhotic Cardiomyopathy in Rats

**DOI:** 10.7759/cureus.c238

**Published:** 2025-08-11

**Authors:** Dlshad Mohammed, Seyed Mohammad Tavangar, Arash Khodadoostan, Seyyedeh Elaheh Mousavi, Ahmad-Reza Dehpour, Farahnaz Jazaeri

**Affiliations:** 1 Pharmacology, Tehran University of Medical Sciences, Tehran, IRN; 2 Pathology, Tehran University of Medical Sciences, Tehran, IRN; 3 Pharmacology, Shahid Beheshti University of Medical Sciences, Tehran, IRN

The reference list of this article has been corrected as follows: The article title in reference 20 has been updated to specify that this article has been retracted. References 44 and 45 were duplicates. Reference 45 has been removed and the in-text citation (at the end of the Discussion section) has been relabeled to cite reference 45.

